# Testis-enriched heat shock protein A2 (HSPA2): Adaptive advantages of the birds with internal testes over the mammals with testicular descent

**DOI:** 10.1038/srep18770

**Published:** 2016-01-06

**Authors:** Abinash Padhi, Mona M. Ghaly, Li Ma

**Affiliations:** 1 Department of Animal and Avian Sciences, University of Maryland, College Park, MD, USA; 2 Department of Animal Production, Faculty of Agriculture, Cairo University, Giza, Egypt

## Abstract

The molecular chaperone heat shock protein A2 (HSPA2), a member of the 70 kDa heat shock protein (HSP70) family, plays an important role in spermatogenesis and male fertility. Although HSPA2 is evolutionarily highly conserved across the metazoan lineages, the observation of striking differences in temperature-sensitive expressions, testicular physiology, spermatogenesis, as well as its role in male fertility indicates that avian and mammalian HSPA2 may exhibit distinct evolutionary trajectory. The present study reports that while mammalian HSPA2 is constrained by intense purifying selection, avian HSPA2 has been subjected to positive selection. The majority of the positively selected amino acid residues fall on the α-helix and β-sheets of the peptide-binding domain located at the carboxyl-terminal region of the avian HSPA2. The detection of positively selected sites at the helix and β-sheets, which are less tolerant to molecular adaptation, indicates an important functional consequence and contribution to the structural and functional diversification of the avian HSPA2. Collectively, avian HSPA2 may have an adaptive advantage over the mammals in response to heat stress, and therefore, mammals with testicular descent may be at a greater risk in the event of scrotal temperature rise.

Spermatogenesis, the most fundamental biological process in male reproductive system, is strongly yet adversely affected by the increase in scrotal temperature in mammals with descended testicle^1^,^2^,^3^,^4^,^5^,^6^,^7^,^8^,^9^. Such increase in scrotal temperature would result in impaired spermatogenesis, thus ultimately affecting the reproductive potential of a wide range of mammalian species with testicular descent, including our own species *Homo sapiens*^1^,^2^,^3^,^5^,^8^. Therefore, in long-term, the elevated surrounding temperature may pose a major threat to the mammalian diversity. While most mammals, with a few exceptions^4^, have external testes where spermatogenesis occurs at 2–8 °C lower than the core body temperatures that range from 35 to 39 °C^2^,^3^,^4^,^6^,^7^,^8^,^10^,^11^,^12^,^13^,^14^, birds maintain an efficient spermatogenesis at an elevated internal body temperature of 40–41 °C 6,13. In contrast to the efficient spermatogenesis at the elevated internal body temperature (40–41 °C) in birds^6^,^13^, mammalian male germ cell was reported to have undergone apoptosis at an internal body temperature of 37 °C 6, thus indicating fundamental differences in spermatogenesis between these two homoeothermic groups^6^,^13^. Recent findings suggest that the testis-enriched heat shock protein A2 (HSPA2), which is reported to exhibit temperature-dependent yet contrasting patterns of expression in mammalian^3^,^15^,^16^ and avian species (e.g.^17^,^18^), play a crucial role in spermatogenesis and male fertility^3^,^19^,^20^,^21^,^22^. Its expression in the testis or in spermatozoa is reported to decrease in men with abnormal spermatogenesis^23^,^24^,^25^,^26^,^27^,^28^. Nevertheless, such striking differences in the mammalian and avian spermatogenesis indicate that HSPA2 may exhibit unique evolutionary trajectory in respective lineages, and the avian species are likely to have an adaptive advantage over the scrotal mammals in response to the elevated surrounding temperature (e.g.^3^,^6^,^15^,^16^,^18^,^29^). Under such circumstances, functional modifications due to the adaptive evolutionary changes of certain amino acid residues in the avian HSPA2 are expected.

The molecular chaperone HSPA2, which is a member of the 70 kDa heat shock protein (HSP70) family^19^,^20^,^22^,^30^,^31^ and is characterized by the presence of an ATPase domain at the N-terminal followed by a peptide binding domain and a G/P-rich domain at the C-terminal^19^,^30^,^32^, regulates the expression of sperm surface receptors involved in sperm-oocyte recognition in humans^19^,^20^,^21^, thus suggesting its vital role in fertility. While HSPA2 was reported to be down-regulated in mammalian male germ cells in response to heat stress^3^,^15^,^16^,^29^, this gene was up-regulated in chicken^18^. Collectively, such contrasting patterns of expression of HSPA2 in birds and mammals in response to acute heat stress further indicate that despite being evolutionarily highly conserved across the metazoan lineages^22^,^31^, this chaperone may exhibit distinct selection profiles in the avian and mammalian lineages.

Given such temperature-sensitive contrasting patterns of expression of this testis-enriched HSPA2 in the avian and mammalian germ cells^3^,^15^,^16^,^18^,^29^, we hypothesize that avian HSPA2 may have experienced positive selection and is therefore likely to have evolved adaptively, whereas the mammalian HSPA2 may have experienced intense purifying selection. By quantifying the ratio of the rates of non-synonymous (amino acid replacement) (dN) and synonymous (no change in amino acid) (dS) substitutions (ω = dN/dS), which has been widely used to detect the footprints of natural selection in the protein-coding genes^33^, we seek to evaluate the pervasive role of positive selection in the evolution of avian and mammalian HSPA2.

## Results and Discussion

**Figure 1:**
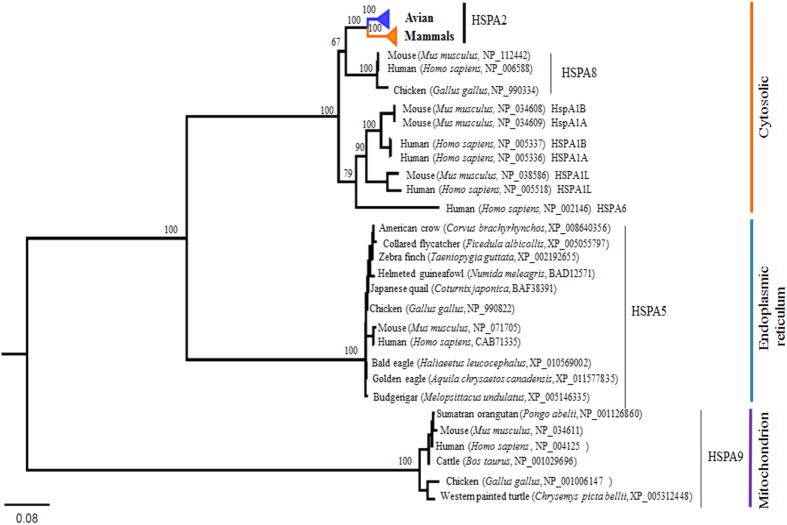
Maximum likelihood (ML) tree inferred from the complete amino acid sequences representing multiple species depicting relationships among the members of HSP70 family. Bootstrap values >70 are shown at the base of the nodes. Common name, scientific name, and accession numbers are shown.

**Figure 2:**
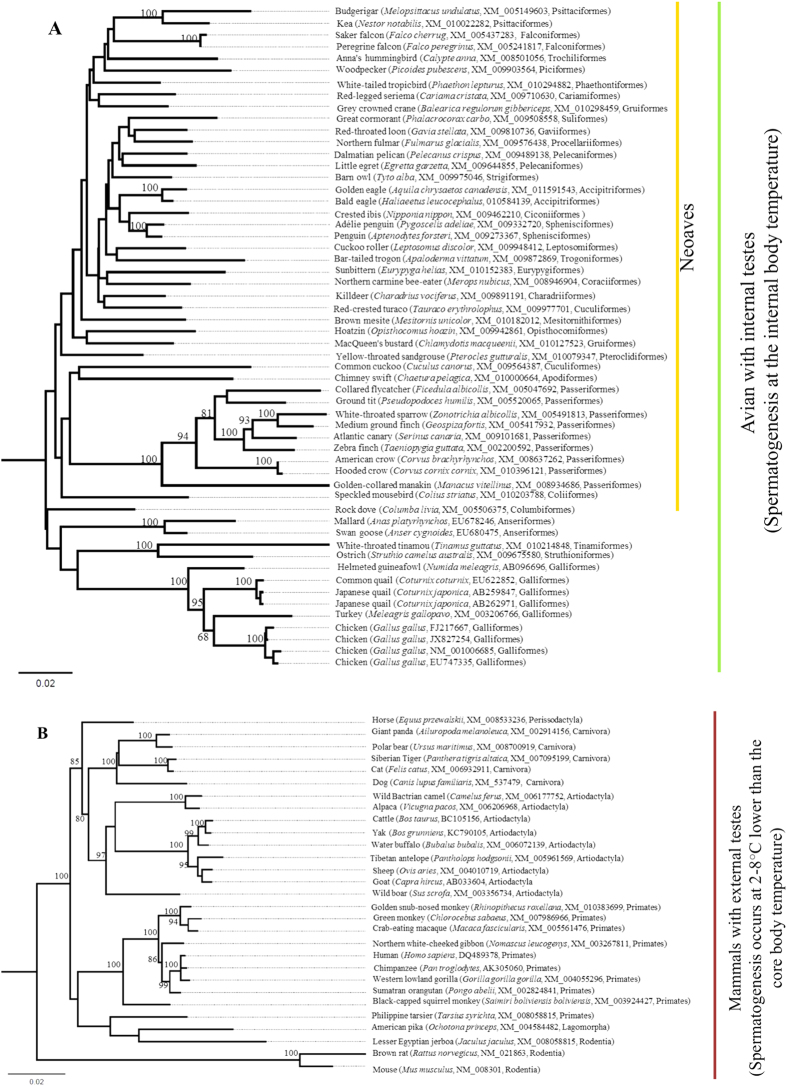
ML trees inferred from the complete nucleotide coding sequence data of (**A**) avian and (**B**) mammalian HSPA2. Bootstrap values >70 are shown at the base of the nodes. Common name, scientific name, nucleotide accession numbers, and systematic order of each species are shown. Spermatogenesis in birds^6^,^13^ and mammals^2^,^4^,^6^,^14^ occurs at the internal body temperature and at 2–8 °C lower than the core body temperature, respectively.

Phylogenetic analyses based on the amino acid sequences representing the members of HSP70 family revealed the orthology and monophyly of the avian and mammalian HSPA2 with strong nodal support (Fig. 1). The phylogenetic affiliations of other members of the family are consistent with the results of a previous study^31^. The avian and mammalian HSPA2 gene trees (Fig. 2) revealed phylogenetic consistency in the placement of each group/order with the previously reported genome-based avian and mammalian phylogenies^34^,^35^,^36^,^37^. However, neither of the gene trees showed habitat/temperature-gradient-based phylogenetic clustering, rather the clusterings are consistent with their systematic classifications (i.e., family; order). Given the long evolutionary history of HSPA2^22^,^31^, the observation of high sequence homologies (and low sequence divergence) of HSPA2 at the amino acid and nucleotide levels between and within the mammalian and avian groups (Table 1; Fig. S1) further indicates that this testis-enriched protein is evolutionarily conserved across the metazoan lineages. Based on this observation, one might speculate that HSPA2 is likely to have been subjected to intense purifying selection throughout the mammalian and avian evolution. However, despite being evolutionarily highly conserved, the observation of striking differences in gene expressions^3^,^15^,^16^,^18^,^29^, testicular physiology, spermatogenesis^4^,^6^,^38^, as well as its crucial role in male fertility^3^,^6^,^19^,^20^,^21^,^22^, prompted us to hypothesize that this testis-enriched HSPA2 may have been subjected to differential selection pressures in these two groups.

Interestingly, while the null models (M1a and M7) that assume no positive selection could not be rejected for the mammalian HSPA2 (*p* > 0.05; Table 2), the corresponding alternative selection models (M2a, M8) are the best-fit models for the avian HSPA2 (*p* < 0.05; Table 2), indicating the pervasive role of positive selection in the evolution of avian HSPA2. Consistently, analyses using other methods provide evidence of positive selection on the avian HSPA2 (Table 3). The differences in the selection profile between mammalian and avian HSPA2 could possibly be associated with the response of HSPA2 to heat stress/temperature. For instance, while exposure of the scrotal mammalian testes to high temperature has been reported to cause impaired spermatogenesis^2^,^5^, birds maintain an efficient spermatogenesis at the elevated internal body temperature^6^,^13^. This indicates that the avian HSPA2, which has been subjected to positive selection, could possibly adapt to elevated temperature, whereas the mammalian HSPA2, which is constrained by purifying selection, may unlikely function efficiently due to prolonged exposure to high temperature. Nixon *et al.*^19^ proposed three possible factors such as (i) genetic mutations in the encoding sequence of *Hspa2* gene, (ii) epigenetic regulation, and (iii) exposure of developing germ cells to oxidative stress, which may be related to impaired spermatogenesis in mammals^19^. However, given the high sequence homology of HSPA2 across the metazoans^19^,^22^,^30^,^31^, increasing incidence of male infertility in human populations due to genetic mutations in the encoding *Hspa2* gene is highly unlikely. The other two explanations of epigenetic regulation and oxidative stress^19^, however, are more plausible explanations for the high incidence of male infertility in the human populations. Prolonged exposure to high temperature, including the prolonged use of laptop computers, was proven to significantly increase scrotal temperature^39^ and therefore, may adversely affect spermatogenesis^1^,^2^,^5^,^8^. Such prolonged exposure to high temperature may be linked to the gene expression and oxidative stress. Additionally, factors such as age^40^,^41^,^42^,^43^,^44^,^45^, exposure to pollutants and individual lifestyle could also affect spermatogenesis^12^. However, how these factors adversely affect the spermatogenesis and the underlying mechanisms need to be explored.

**Figure 3:**
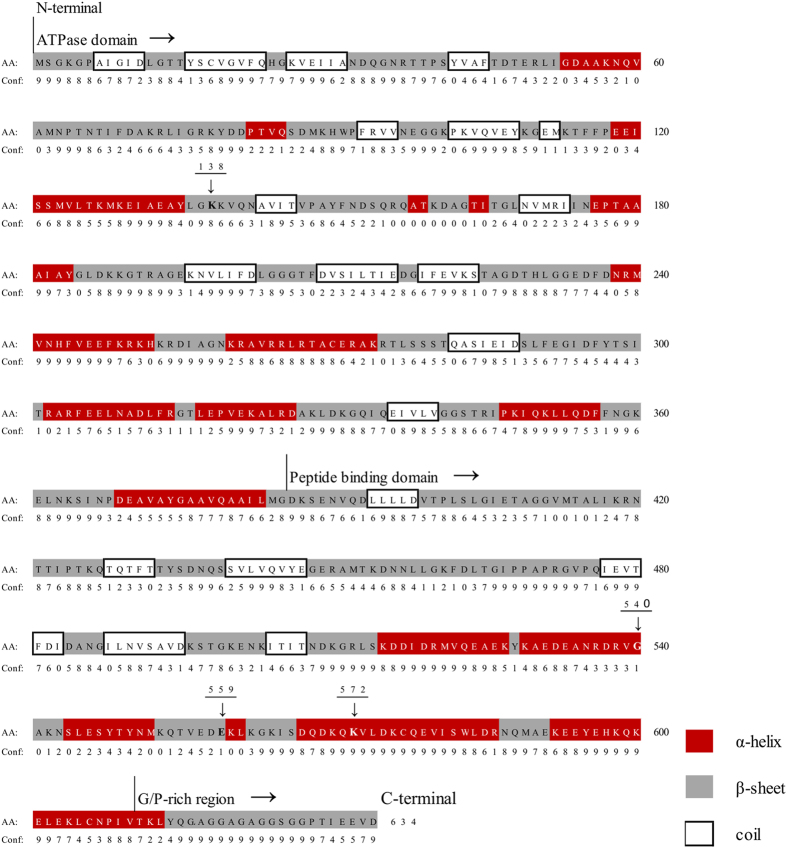
Predicted secondary structure and functional domains of chicken HSPA2. ATPase domain (~385 amino acids), peptide binding domain (~225 amino acids), and G/P-rich region (~30 amino acids) are shown. Predicted domains (α-helix, β-sheet, and coil) and their respective confidence values (0, low; 9, high) are also shown. Two of the positively selected sites (site-540 and site-572) are predicted to be in the α-helix located at the peptide binding domain, whereas site-559 is in the coil of the peptide binding domain. Site-138 is in the coil at the ATPase domain. Secondary structure was predicted by using the PSIPRED server^58^. Functional domains were identified based on the previously published reports^30^,^32^.

Taken together with the results of previous studies^2^,^5^,^6^, our study indicates that the observed adaptive evolutionary changes in certain amino acid residues of the avian HSPA2 are likely temperature-driven. Of the four amino acid residues that were detected to be under positive selection in the avian HSPA2 (Table 3), three residues occur close to one another and are located at the end of the gene (i.e., C-terminal), a pattern that is consistent with previous studies^46^. Further, given the fact that protein secondary structure has a significant effect on the rate of protein adaptation^46^,^47^, we sought to find the location of the positively selected sites in the avian HSPA2 protein secondary structure. Interestingly, while three sites (sites 540, 559, and 572) are located at the peptide binding domain, only one site (site 138) is located at the ATPase domain. Of the three sites at the peptide binding domain, site-540 and site-572 are on the α-helix with high confidence, and site-559 falls at the β-sheet, however, with low confidence (Fig. 3). While detections of positively selected sites at the coil regions are common^46^, the detections of positively selected sites at the helix and β-sheets, which are less tolerant to molecular adaptation, are less common^46^. However, if any of the residues in the helix and β structures were detected to be under positive selection, the protein may have important functional consequences^46^. Under this circumstance, the avian HSPA2 may have exhibited functional diversification driven by the positive Darwinian selection. Intuitively, although the specific roles of these three positively selected amino acid residues located at the peptide binding domain are unknown, given the regulatory role of HSPA2 in sperm-oocyte recognition in humans^19^,^20^,^21^, these amino acid residues in avian HSPA2 may have important functional significance. Nevertheless, these predictions warrant further investigations on the biological significance of these positively selected residues in relation to temperature/heat adaptation.

Collectively, the present study indicates that while mammalian HSPA2 has been constrained by purifying selection, the avian HSPA2 has been subjected to positive selection and therefore, has an adaptive advantage over the mammalian HSPA2. Mammals with testicular descent, including our own species *Homo sapiens*, therefore, is at a greater risk in the event of prolonged exposure of the testicle to high temperature, as it would ultimately affect the spermatogenesis. However, further studies are required to explore the possible involvement of multiple testis-specific temperature-dependent genes that affect spermatogenesis and male fertility, their expression patterns in response to heat stress as well as how natural selection has shaped the evolution of those genes (if any) in the avian and mammalian groups.

## Methods

Complete nucleotide coding sequences of the avian and mammalian HSPA2 as well as the complete nucleotide and amino acid sequences of other members of the HSP70 family were retrieved from GenBank^48^. Amino acid and nucleotide coding sequences were aligned using the MUSCLE algorithm implemented in MEGA ver. 5 49. To determine the phylogenetic relatedness and monophyly of the avian and mammalian HSPA2, we reconstructed maximum likelihood (ML)-based HSP70 phylogeny using the previously reported complete amino acid sequences representing the members of the HSP70 family as reference sequences^31^. The accession numbers of the amino acid sequences that were used as reference sequences for the respective members are shown in Fig. 1. The best-fit amino acid substitution model was selected by the Bayesian Information Criterion (BIC) implemented in MEGA 5^49^. The amino acid based HSP70 ML phylogeny was reconstructed under the JTT (Jones-Taylor-Thornton) model with gamma distribution shape parameter (G) using MEGA 5^49^. Using the same program nodal supports were estimated with 1000 bootstrap replicates. To assess the strength of natural selection on the avian and mammalian HSPA2, we retrieved respectively 56 avian HSPA2 complete nucleotide coding sequences representing 52 species, 41 families and 31 orders, as well as 29 mammalian HSPA2 complete nucleotide coding sequences representing 29 species, 16 families, and 6 orders from GenBank^48^. The nucleotide accession numbers of the avian and mammalian HSPA2 used in the present study are shown in Fig. 2A,B. The evolutionary divergence between the avian and mammalian groups and within each group was estimated using the MEGA program^49^. Standard errors of the distance estimates were estimated with 1000 replicates. Using the PhyML ver 3 50 program, ML-based mammalian and avian HSPA2 phylogenies were constructed under the best-fit nucleotide substitution models of the respective data sets. The best-fit nucleotide substitution models for the respective data sets were selected by BIC implemented in jModelTest2 51. Phylogenetic trees were visualized using the FigTree 1.4.2 software (available at http://tree.bio.ed.ac.uk/software/figtree/).

The test for positive selection was performed using the ML based codon substitution models^33^ implemented in the CODEML program of PAML package^52^. The likelihood ratio test (LRT) was used to compare the null models (M1a and M7) that assume no positive selection (ω < 1) with their corresponding alternative models (M2a and M8) that assume positive selection (ω > 1), respectively^33^. Additionally, sites under positive selection were also detected using the SLAC (single-likelihood ancestor counting), FEL (fixed effects likelihood), IFEL (internal fixed effects likelihood), REL (random effects likelihood), and FUBAR (fast unbiased Bayesian approximation) methods^53^,^54^,^55^ implemented in the datamonkey server^56^.

Secondary structure predictions and the confidence values for the avian HSPA2 protein were made by using the PSIPRED^57^ and the Protein Structure Prediction Server (http://bioinf.cs.ucl.ac.uk/psipred)^58^.

## Additional Information

**How to cite this article**: Padhi, A. *et al.* Testis-enriched heat shock protein A2 (HSPA2): Adaptive advantages of the birds with internal testes over the mammals with testicular descent. *Sci. Rep.* **6**, 18770; doi: 10.1038/srep18770 (2016).

## Supplementary Material

Supplementary Information

## Figures and Tables

**Table 1:** Estimates of net evolutionary divergence (in %) between avian and mammalian groups and within each group.

Sequence	Between group (Mammal–Avian)	Within group	
		Mammal	Avian
DNA	21.63 ± 1.87	6.81 ± 0.39	8.35 ± 0.48
Amino acid	6.08 ± 0.83	0.72 ± 0.17	1.12 ± 0.23

**Table 2:** Likelihood Ratio Tests (LRTs) statistic for positive selection on the avian and mammalian HSPA2.

Model comparison	Avian					Mammal				
	Parameters					Parameters				
	*2*Δ*l*	p-value	Positively selected site	Pr(ω > 1)	ω (±SE)	*2*Δ*l*	p-value	Positively selected site	Pr (ω>1)	ω (±SE)
M1a *vs* M2a	172.22	4E-38	540 G	0.82	1.71 ± 0.59	0.00	1.00	None	None	None
			572 K	0.85	1.72 ± 0.56					
M7 *vs* M8	64.34	1E-14	540 G	0.99	1.69 ± 0.43	8.76E-04	1.00	None	None	None
			572 K	0.99	1.70 ± 0.42					

Position of the positively selected amino acid sites are corresponding to the position of the amino acid sites in the chicken amino acid sequence (GenBank accession number: AFX69291).

Null models (no positive selection): M1a, M7; Alternative models (positive selection): M2a, M8.

ω: Number of nonsynonymous substitutions per nonsynonymous site (dN)/ Number of synonymous substitutions per synonymous site (dS).

Pr(ω>1): Probability of having ω > 1.

SE: Standard error.

Δl: Differences in the likelihood scores between null and alternative models.

Degrees of freedom for model comparison: 2.

**Table 3:** Positively selected sites detected in the avian and mammalian HSPA2 under different methods.

Group	Overall ω	SLAC	FEL	IFEL	REL	FUBAR	PAML (M8 model)
Avian	0.018 (0.014–0.022)	**559**	**138**, **559**	**138**, **559**	3, 99, **540**, **559**, **572**	**138**, **559**, **572**	**540**, **572**
Mammal	0.015 (0.011–0.020)	None	None	None	None	None	None

Sites detected to be under positive selection by more than one method are in bold.

ω: Number of nonsynonymous substitutions per nonsynonymous site (dN)/ Number of synonymous substitutions per synonymous site (dS).

SLAC: Single Likelihood Ancestral Counting, FEL: Fixed Effects Likelihood, IFEL: Internal Fixed Effects Likelihood, REL: Random Effects Likelihood. FUBAR: Fast Unconstrained Bayesian Approximation, PAML: Phylogenetic Analysis by Maximum Likelihood.

## References

[b1] Durairajanayagam D., Agarwal A. & Ong C. Causes, effects and molecular mechanisms of testicular heat stress. Reprod. Biomed. Online 30, 14–27 (2015). 25456164 10.1016/j.rbmo.2014.09.018

[b2] Hansen P. J. Effects of heat stress on mammalian reproduction. Phil. Trans. R. Soc. Lond. B Biol.Sci. 364, 3341–3350 (2009). 19833646 10.1098/rstb.2009.0131 PMC2781849

[b3] Kim B., Park K. & Rhee K. Heat stress response of male germ cells. Cell. Mol. Life Sci. 70, 2623–2636 (2013). 23007846 10.1007/s00018-012-1165-4 PMC11113252

[b4] Kleisner K., Ivell R. & Flegr J. The evolutionary history of testicular externalization and the origin of the scrotum. J. Biosci. 35, 27–37 (2010). 20413907 10.1007/s12038-010-0005-7

[b5] Kong W. H., Zheng G., Lu J. N. & Tso J. K. Temperature dependent expression of cdc2 and cyclin B1 in spermatogenic cells during spermatogenesis. Cell Res. 10, 289–302 (2000). 11191351 10.1038/sj.cr.7290056

[b6] Mezquita B., Mezquita C. & Mezquita J. Marked differences between avian and mammalian testicular cells in the heat shock induction and polyadenylation of Hsp70 and ubiquitin transcripts. FEBS Lett. 436, 382–386 (1998). 9801153 10.1016/s0014-5793(98)01172-7

[b7] Prontera P. & Donti E. Hypothesis: gonadal temperature influences sex-specific imprinting. Front. Genet. 5, 294 (2014). 25202325 10.3389/fgene.2014.00294 PMC4142806

[b8] Setchell B. P. The Parkes Lecture. Heat and the testis. J. Reprod. Fertil. 114, 179–194 (1998). 10070346 10.1530/jrf.0.1140179

[b9] Skinner J. D. & Louw G. N. Heat stress and spermatogenesis in *Bos indicus* and *Bos taurus* cattle. J. Appl. Physiol. 21, 1784–1790 (1966). 5951717 10.1152/jappl.1966.21.6.1784

[b10] Brito L. F., Silva A. E., Barbosa R. T. & Kastelic J. P. Testicular thermoregulation in *Bos indicus*, crossbred and *Bos taurus* bulls: relationship with scrotal, testicular vascular cone and testicular morphology, and effects on semen quality and sperm production. Theriogenology 61, 511–528 (2004). 14662148 10.1016/s0093-691x(03)00231-0

[b11] Prosser C. L. & Heath J. E. *Temperature*. In Comparative animal physiology, environmental and metabolic animal physiology (ed. Prosser C. L.), pp. 109–166,. 4th edn, 109-166 (John Wiley & Sons, 1991).

[b12] Sharpe R. M. Environmental/lifestyle effects on spermatogenesis. Philos. Trans. R. Soc. Lond. B Biol. Sci. 365, 1697–1712 (2010). 20403879 10.1098/rstb.2009.0206 PMC2871918

[b13] Beaupre C. E. *et al.* Determination of testis temperature rhythms and effects of constant light on testicular function in the domestic fowl (*Gallus domesticus*). Biol. Reprod. 56, 1570–1575 (1997). 9166712 10.1095/biolreprod56.6.1570

[b14] Knobil E. & Neill J. D. The Physiology of Reproduction. 1–1372 (Raven Press, 1995).

[b15] Kon Y. & Endoh D. Heat-shock resistance in experimental cryptorchid testis of mice. Mol. Reprod. Dev. 58, 216–222 (2001). 11139234 10.1002/1098-2795(200102)58:2<216::AID-MRD11>3.0.CO;2-C

[b16] Widlak W. *et al.* Heat shock transcription factor 1 down-regulates spermatocyte-specific 70 kDa heat shock protein expression prior to the induction of apoptosis in mouse testes. Genes Cells 12, 487–499 (2007). 17397396 10.1111/j.1365-2443.2007.01069.x

[b17] Wang S. H. *et al.* Differential gene expressions in testes of L2 strain Taiwan country chicken in response to acute heat stress. Theriogenology 79, 374–382 e371–377 (2013). 10.1016/j.theriogenology.2012.10.010 23154143

[b18] Wang S. H. *et al.* Acute heat stress induces differential gene expressions in the testes of a broiler-type strain of taiwan country chickens. PloS ONE 10, e0125816 (2015). 25932638 10.1371/journal.pone.0125816 PMC4416790

[b19] Nixon B. *et al.* The role of the molecular chaperone heat shock protein A2 (HSPA2) in regulating human sperm-egg recognition. Asian J. Androl. 17, 568–573 (2015). 25865850 10.4103/1008-682X.151395 PMC4492046

[b20] Redgrove K. A. *et al.* Investigation of the mechanisms by which the molecular chaperone HSPA2 regulates the expression of sperm surface receptors involved in human sperm-oocyte recognition. Mol. Hum. Reprod. 19, 120–135 (2013). 23247813 10.1093/molehr/gas064

[b21] Redgrove K. A. *et al.* The molecular chaperone HSPA2 plays a key role in regulating the expression of sperm surface receptors that mediate sperm-egg recognition. PloS ONE 7, e50851 (2012). 23209833 10.1371/journal.pone.0050851 PMC3510172

[b22] Scieglinska D. & Krawczyk Z. Expression, function, and regulation of the testis-enriched heat shock HSPA2 gene in rodents and humans. Cell Stress Chaperones 20, 221–235 (2015). 25344376 10.1007/s12192-014-0548-x PMC4326386

[b23] Cedenho A. P. *et al.* Oligozoospermia and heat-shock protein expression in ejaculated spermatozoa. Hum. Reprod. 21, 1791–1794 (2006). 16517558 10.1093/humrep/del055

[b24] Feng H. L., Sandlow J. I. & Sparks A. E. Decreased expression of the heat shock protein hsp70-2 is associated with the pathogenesis of male infertility. Fertil. Steril. 76, 1136–1139 (2001). 11730740 10.1016/s0015-0282(01)02892-8

[b25] Lima S. B. *et al.* Expression of the HSPA2 gene in ejaculated spermatozoa from adolescents with and without varicocele. Fertil. Steril. 86, 1659–1663 (2006). 17007855 10.1016/j.fertnstert.2006.05.030

[b26] Motiei M., Tavalaee M., Rabiei F., Hajihosseini R. & Nasr-Esfahani M. H. Evaluation of HSPA2 in fertile and infertile individuals. Andrologia 45, 66–72 (2013). 22670834 10.1111/j.1439-0272.2012.01315.x

[b27] Son W. Y. *et al.* Repression of hspA2 messenger RNA in human testes with abnormal spermatogenesis. Fertil. Steril. 73, 1138–1144 (2000). 10856471 10.1016/s0015-0282(00)00496-9

[b28] Terribas E. *et al.* Changes in the expression profile of the meiosis-involved mismatch repair genes in impaired human spermatogenesis. J. Androl. 31, 346–357 (2010). 20075417 10.2164/jandrol.109.008805

[b29] Zhou X. C., Han X. B., Hu Z. Y., Zhou R. J. & Liu Y. X. Expression of Hsp70-2 in unilateral cryptorchid testis of rhesus monkey during germ cell apoptosis. Endocrine 16, 89–95 (2001). 11887939 10.1385/ENDO:16:2:089

[b30] Daugaard M., Rohde M. & Jaattela M. The heat shock protein 70 family: Highly homologous proteins with overlapping and distinct functions. FEBS Lett. 581, 3702– 3710 (2007). 17544402 10.1016/j.febslet.2007.05.039

[b31] McCallister C., Siracusa M. C., Shirazi F., Chalkia D. & Nikolaidis N. Functional diversification and specialization of cytosolic 70-kDa heat shock proteins. Sci. Rep. 5 (2015). 10.1038/srep09363 PMC4366816 25791537

[b32] Hartl F. U. Molecular chaperones in cellular protein folding. Nature 381, 571–579 (1996). 8637592 10.1038/381571a0

[b33] Yang Z., Nielsen R., Goldman N. & Pedersen A. M. Codon-substitution models for heterogeneous selection pressure at amino acid sites. Genetics 155, 431–449 (2000). 10790415 10.1093/genetics/155.1.431 PMC1461088

[b34] Jarvis E. D. *et al.* Whole-genome analyses resolve early branches in the tree of life of modern birds. Science 346, 1320–1331 (2014). 25504713 10.1126/science.1253451 PMC4405904

[b35] McCormack J. E. *et al.* A phylogeny of birds based on over 1,500 loci collected by target enrichment and high-throughput sequencing. PloS ONE 8, e54848 (2013). 23382987 10.1371/journal.pone.0054848 PMC3558522

[b36] Zhang G. *et al.* Comparative genomics reveals insights into avian genome evolution and adaptation. Science 346, 1311–1320 (2014). 25504712 10.1126/science.1251385 PMC4390078

[b37] Song S., Liu L., Edwards S. V. & Wu S. Resolving conflict in eutherian mammal phylogeny using phylogenomics and the multispecies coalescent model. Proc. Natl. Acad. Sci. USA 109, 14942–14947 (2012). 22930817 10.1073/pnas.1211733109 PMC3443116

[b38] Jones R. C. & Lin M. Spermatogenesis in birds. Oxf. Rev. Reprod. Biol. 15, 233–264 (1993). 8336978

[b39] Sheynkin Y., Jung M., Yoo P., Schulsinger D. & Komaroff E. Increase in scrotal temperature in laptop computer users. Hum. Reprod. 20, 452–455 (2005). 15591087 10.1093/humrep/deh616

[b40] Angelopoulou R., Lavranos G., Manolakou P. & Katsiki E. Fertility in the aging male: molecular pathways in the anthropology of aging. Coll. Antropol. 37, 657–661 (2013). 23941021

[b41] Harris I. D., Fronczak C., Roth L. & Meacham R. B. Fertility and the aging male. Rev. Urol. 13, e184–190 (2011). 22232567 PMC3253726

[b42] Matorras R., Matorras F., Exposito A., Martinez L. & Crisol L. Decline in human fertility rates with male age: a consequence of a decrease in male fecundity with aging? Gynecol. Obstet. Invest. 71, 229–235 (2011). 21160151 10.1159/000319236

[b43] Plas E., Berger P., Hermann M. & Pfluger H. Effects of aging on male fertility? Exp. Gerontol. 35, 543–551 (2000). 10978677 10.1016/s0531-5565(00)00120-0

[b44] Purandhar K., Jena P. K., Prajapati B., Rajput P. & Seshadri S. Understanding the role of heat shock protein isoforms in male fertility, aging and apoptosis. World J. Mens Health 32, 123–132 (2014). 25606560 10.5534/wjmh.2014.32.3.123 PMC4298814

[b45] Stewart A. F. & Kim E. D. Fertility concerns for the aging male. Urology 78, 496–499 (2011). 21884897 10.1016/j.urology.2011.06.010

[b46] Ridout K. E., Dixon C. J. & Filatov D. A. Positive selection differs between protein secondary structure elements in Drosophila. Genome Biol. Evol. 2, 166–179 (2010). 20624723 10.1093/gbe/evq008 PMC2997536

[b47] Sawyer S. L., Wu L. I., Emerman M. & Malik H. S. Positive selection of primate TRIM5alpha identifies a critical species-specific retroviral restriction domain. Proc. Natl. Acad. Sci. USA 102, 2832–2837 (2005). 15689398 10.1073/pnas.0409853102 PMC549489

[b48] Benson D. A. *et al.* GenBank. Nucleic Acids Res. 43, D30–35 (2015). 25414350 10.1093/nar/gku1216 PMC4383990

[b49] Tamura K. *et al.* MEGA5: molecular evolutionary genetics analysis using maximum likelihood, evolutionary distance, and maximum parsimony methods. Mol. Biol. Evol. 28, 2731–2739 (2011). 21546353 10.1093/molbev/msr121 PMC3203626

[b50] Guindon S. & Gascuel O. A simple, fast, and accurate algorithm to estimate large phylogenies by maximum likelihood. Syst. Biol. 52, 696–704 (2003). 14530136 10.1080/10635150390235520

[b51] Darriba D., Taboada G. L., Doallo R. & Posada D. jModelTest 2: more models, new heuristics and parallel computing. Nat. Methods 9, 772 (2012). 22847109 10.1038/nmeth.2109 PMC4594756

[b52] Yang Z. PAML 4: phylogenetic analysis by maximum likelihood. Mol. Biol. Evol. 24, 1586–1591 (2007). 17483113 10.1093/molbev/msm088

[b53] Kosakovsky Pond S. L. & Frost S. D. Not so different after all: a comparison of methods for detecting amino acid sites under selection. Mol. Biol. Evol. 22, 1208–1222 (2005). 15703242 10.1093/molbev/msi105

[b54] Murrell B. *et al.* FUBAR: a fast, unconstrained bayesian approximation for inferring selection. Mol. Biol. Evol. 30, 1196–1205 (2013). 23420840 10.1093/molbev/mst030 PMC3670733

[b55] Pond S. L. *et al.* Adaptation to different human populations by HIV-1 revealed by codon-based analyses. PLoS Comput. Biol. 2, e62 (2006). 16789820 10.1371/journal.pcbi.0020062 PMC1480537

[b56] Pond S. L. & Frost S. D. Datamonkey: rapid detection of selective pressure on individual sites of codon alignments. Bioinformatics 21, 2531–2533 (2005). 15713735 10.1093/bioinformatics/bti320

[b57] Jones D. T. Protein secondary structure prediction based on position-specific scoring matrices. J. Mol. Biol. 292, 195–202 (1999). 10493868 10.1006/jmbi.1999.3091

[b58] Buchan D. W., Minneci F., Nugent T. C., Bryson K. & Jones D. T. Scalable web services for the PSIPRED Protein Analysis Workbench. Nucleic Acids Res. 41, W349–357 (2013). 23748958 10.1093/nar/gkt381 PMC3692098

