# Supplementary material for: Testis-enriched heat shock protein A2 (HSPA2): Adaptive advantages of the birds with internal testes over the mammals with testicular descent
Source: Sci Rep. 2016 Jan 6;6:18770. doi: 10.1038/srep18770 (PMC4702119; doi:10.1038/srep18770)
Supplement: Supplementary Information [file srep18770-s1.pdf]

**Title: Testis-enriched heat shock protein A2 (HSPA2): Adaptive advantages of the birds with internal testes over the mammals with testicular descent**

**Authors:**Abinash Padhi, Mona M. Ghaly, Li Ma

**Supp. Fig. S1.** Amino acid sequence alignment of human HSPA2 with mouse, chicken and Guineafowl HSPA2. Variable amino acid sites are highlighted in gray. All the three functional domains are shown. Site that were detected to be under positive selection in Avian HSPA2 are marked with asterisk.

```
ATPase domain
Human (ABE96830)       MSARGPAIGIDLGTTYSCVGVFQHGKVEIIANDQGNRTTPSYVAFTDTERLIGDAAKNQVAMNPTNTIFDAKRLIGRKFE [ 80]
Mouse (NP_032327)      MSARGPAIGIDLGTTYSCVGVFQHGKVEIIANDQGNRTTPSYVAFTDTERLIGDAAKNQVAMNPTNTIFDAKRLIGRKFE [ 80]
Chicken (AFX69291)     MSGKGPAIGIDLGTTYSCVGVFQHGKVEIIANDQGNRTTPSYVAFTDTERLIGDAAKNQVAMNPTNTIFDAKRLIGRKYD [ 80]
Guineafowl (BAC24791)  MSGKGPAIGIDLGTTYSCVGVFQHGKVEIIANDQGNRTTPSYVAFTDTERLIGDAAKNQVAMNPTNTIFDAKRLIGRKYD [ 80]

Human (ABE96830)       DATVQSDMKHWPFRVVSEGGKPKVQVEYKGETKTFFPEEISSMVLTKMKEIAEAYLGGKVHSAVITVPAYFNDSQRQATK [160]
Mouse (NP_032327)      DATVQSDMKHWPFRVVSEGGKPKVQVEYKGEMKTFFPEEISSMVLTKMKEIAEAYLGGKVQSAVITVPAYFNDSQRQATK [160]
Chicken (AFX69291)     DPTVQSDMKHWPFRVVNEGGKPKVQVEYKGEMKTFFPEEISSMVLTKMKEIAEAYLGKKVQNAVITVPAYFNDSQRQATK [160]
Guineafowl (BAC24791)  DPTVRSDMKHWPFRVVNEGGKPKAQVEYKGEMKTFFPEEISSMVLTKMKEIAEAYLGKKVQNAVITVPAYFNDSQRQATK [160]

Human (ABE96830)       DAGTITGLNVLRIINEPTAAAIAYGLDKKGCAGGEKNVLIFDLGGGTFDVSILTIEDGIFEVKSTAGDTHLGGEDFDNRM [240]
Mouse (NP_032327)      DAGTITGLNVLRIINEPTAAAIAYGLDKKGCAGGEKNVLIFDLGGGTFDVSILTIEDGIFEVKSTAGDTHLGGEDFDNRM [240]
Chicken (AFX69291)     DAGTITGLNVMRIINEPTAAAIAYGLDKKGTRAGEKNVLIFDLGGGTFDVSILTIEDGIFEVKSTAGDTHLGGEDFDNRM [240]
Guineafowl (BAC24791)  DAGTITGLNVMRIINEPTAAAIAYGLDKKGTRAGEKNVLIFDLGGGTFDVSILTIEDGIFEVKSTAGDTHLGGEDFDNRM [240]

Human (ABE96830)       VSHLAEEFKRKHKKDIGPNKRAVRRLRTACERAKRTLSSSTQASIEIDSLYEGVDFYTSITRARFEELNADLFRGTLEPV [320]
Mouse (NP_032327)      VSHLAEEFKRKHKKDIGPNKRAVRRLRTACERAKRTLSSSTQASIEIDSLYEGVDFYTSITRARFEELNADLFRGTLEPV [320]
Chicken (AFX69291)     VNHFVEEFKRKHKRDIAGNKRAVRRLRTACERAKRTLSSSTQASIEIDSLFEGIDFYTSITRARFEELNADLFRGTLEPV [320]
Guineafowl (BAC24791)  VNHFVEEFKRKHKRDIAGNKRAVRRLRTACERAKRTLSSSTQASIEIDSLFEGIDFYTSITRARFEELNADLFRGTLEPV [320]
                                                                                   Peptide binding domain
Human (ABE96830)       EKALRDAKLDKGQIQEIVLVGGSTRIPKIQKLLQDFFNGKELNKSINPDEAVAYGAAVQAAILIGDKSENVQDLLLLDVT [400]
Mouse (NP_032327)      EKALRDAKLDKGQIQEIVLVGGSTRIPKIQKLLQDFFNGKELNKSINPDEAVAYGAAVQAAILIGDKSENVQDLLLLDVT [400]
Chicken (AFX69291)     EKALRDAKLDKGQIQEIVLVGGSTRIPKIQKLLQDFFNGKELNKSINPDEAVAYGAAVQAAILMGDKSENVQDLLLLDVT [400]
Guineafowl (BAC24791)  EKALRDAKLDKGQIQEIVLVGGSTRIPKIQKLLQDFFNGKELNKSINPDEAVAYGAAVQAAILMGDKSENVQDLLLLDVT [400]

Human (ABE96830)       PLSLGIETAGGVMTPLIKRNTTIPTKQTQTFTTYSDNQSSVLVQVYEGERAMTKDNNLLGKFDLTGIPPAPRGVPQIEVT [480]
Mouse (NP_032327)      PLSLGIETAGGVMTPLIKRNTTIPTKQTQTFTTYSDNQSSVLVQVYEGERAMTKDNNLLGKFDLTGIPPAPRGVPQIEVT [480]
Chicken (AFX69291)     PLSLGIETAGGVMTALIKRNTTIPTKQTQTFTTYSDNQSSVLVQVYEGERAMTKDNNLLGKFDLTGIPPAPRGVPQIEVT [480]
Guineafowl (BAC24791)  PLSLGIETAGGVMTALIKRNTTIPTKQTQTFTTYSDNQSSVLVQVYEGERAMTKDNNLLGKFDLTGIPPAPCGVPQIEVT [480]

Human (ABE96830)       FDIDANGILNVTAADKSTGKENKITITNDKGRLSKDDIDRMVQEAERYKSEDEANRDRVAAKNALESYTYNIKQTVEDEK [560]
Mouse (NP_032327)      FDIDANGILNVTAADKSTGKENKITITNDKGRLSKDDIDRMVQEAERYKSEDEANRDRVAAKNAVESYTYNIKQTVEDEK [560]
Chicken (AFX69291)     FDIDANGILNVSAVDKSTGKENKITITNDKGRLSKDDIDRMVQEAEKYKAEDEANRDRVGAKNSLESYTYNMKQTVEDEK [560]
Guineafowl (BAC24791)  FDIDANGILNVSAVDKSTGKENKITITNDKGRLSKDDIDRMVQEAEKYKAEDEANRDRVGAKNSLESYTYNMKQTVEDDK [560]
                                                                   G/P-rich region
Human (ABE96830)       LRGKISEQDKNKILDKCQEVINWLDRNQMAEKDEYEHKQKELERVCNPIISKLYQGGPGGGSGGGGSGASGGPTIEEVD [639]
Mouse (NP_032327)      LRGKISEQDKNKILDKCQEVINWLDRNQMAEKDEYEHKQKELERVCNPIISKLYQGGPGGG-----GSSGGPTIEEVD [639]
Chicken (AFX69291)     LKGKISDQDKQKVLDKCQEVISWLDRNQMAEKEEYEHKQKELEKLCNPIVTKLYQGAGGAG-----AGGSGGPTIEEVD [639]
Guineafowl (BAC24791)  LKGKISDQDKQKVLDKCREVISWLDRNQMAEKEEYEHKQKELEKLCNPIVTKLYQGAGGAG-----AGGSGGPTIEEVD [639]
```
